# Helical Piezoelectric Energy Harvester and Its Application to Energy Harvesting Garments

**DOI:** 10.3390/mi8040115

**Published:** 2017-04-04

**Authors:** Minsung Kim, Kwang-Seok Yun

**Affiliations:** Department of Electronic Engineering, Sogang University, Seoul 04107, South Korea; msungkim@lginnotek.com

**Keywords:** piezoelectric energy harvester, helical, energy harvesting garment, human body motions, wearable

## Abstract

In this paper, we propose a helical piezoelectric energy harvester, examine its application to clothes in the form of an energy harvesting garment, and analyze its design and characteristics. The helical harvester is composed of an elastic core and a polymer piezoelectric strap twining the core. The fabricated harvester is highly elastic and can be stretched up to 158% of its initial length. Following the experiments using three different designs, the maximum output power is measured as 1.42 mW at a 3 MΩ load resistance and 1 Hz motional frequency. The proposed helical harvesters are applied at four positions of stretchable tight-fitting sportswear, namely shoulder, arm joint, knee, and hip. The maximum output voltage is measured as more than 20 V from the harvester at the knee position during intended body motions. In addition, electric power is also generated from this energy harvesting garment during daily human motions, which is about 3.9 V at the elbow, 3.1 V at the knee, and 4.4 V at the knee during push-up, walking, and squatting motions, respectively.

## 1. Introduction

Recently, there has been an increasing interest in wearable sensors and it is expected that various smart sensors in wearable form will be used in the near future [1]. Even though these wearable sensors have been widely studied by researchers around the world [2,3,4,5,6], the requirement of a power supply is still an unsolved issue for stable and long term operation of the devices. Currently, a battery is commonly used for a wearable sensor, however, the limited lifetime and need for periodic recharge are its inherent problems.

Energy harvesting is considered as an alternative technology to overcome the issues of the current power supply source for wearable sensors [7,8,9]. An energy harvester utilizes various energy sources to generate electric power, including light [10,11], radio frequency [12,13], temperature difference [14,15,16], and mechanical energy [17,18,19,20,21,22]. Among them, mechanical energy is considered suitable for wearable applications because it is not influenced by environmental conditions such as weather and location.

A wearable piezoelectric energy harvester, based on the piezoelectric transduction mechanism, utilizing human motion as an energy source has been widely researched recently [23,24,25,26,27,28,29]. An energy harvester generating electric power from heel strikes and strain applied on a backpack strap during walking was reported to produce a maximum power of 45.6 mW from the backpack strap [24]. A knee-joint wearable piezoelectric energy harvester was demonstrated by Pozzi et al. [26]. In addition, piezoelectric harvesters in textile structures have been reported by Song et al. and Ahn et al. It was claimed that a peak output power of 1.1 mW was achieved by using woven piezoelectric fabrics [8,29]. Electrospun polyvinylidene fluoride-trifluoroethylene (PVDF-TrFE) fibers are also used to form a stretchable textile by combining coiled piezoelectric fibers and polyurethane fibers [30]. Soin et al. have produced a 3D knitted spacer piezo fabric using melt-spun piezoelectric yarns and silver-coated polyamide yarns [31]. However, the application of a flexible and stretchable piezoelectric harvester to human clothes such as shirts and pants has not been reported until recently [32]. Yang et al. proposed a garment for piezoelectric harvesting from joint motion and showed that the harvesting efficiency was affected by the tightness level depending upon the property of the textile material and design configuration of the garment [25]. 

Previously, we have reported a helical structure piezoelectric energy harvester that can be applied to stretching human motion [27]. The proposed device was very flexible, stretchable, and produced reasonable output power even under slow motion. The measured power was 0.3 mW/cm^3^ at 4 Hz. However, there have not been sufficient studies regarding the helical structure when it is applied to a garment and operated by real human motions. Therefore, in this work, we report the application of helical piezoelectric devices to commercial clothes and characterize these energy harvesting garments by investigating the harvested output power from different positions under various human activities such as walking and running.

## 2. Principle

**Figure 1:**
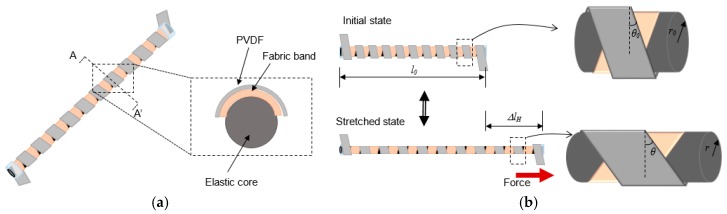
(**a**) Schematic of the proposed helical piezoelectric energy harvester and (**b**) its operation when a stretching force is applied.

Figure 1 shows the schematic of the proposed helical piezoelectric energy harvester (HPEH) and its operation based on a stretching motion. The basic design of the energy harvester, consisting of an elastic core, a fabric band, and a piezoelectric strap, and its operation have been introduced in our previous work [27]. A polyvinylidene fluoride (PVDF) thin film is used as the piezoelectric strap in this work. The PVDF strap and fabric band form two helical structures in counter directions around the elastic core. When the HPEH is stretched as shown in Figure 1b, the helical structure experiences a torsional stress and longitudinal tensile stress toward the pulling direction. Thus, the PVDF helix produces an alternating potential during continuous stretching and contraction motions. A core is formed in the non-overlapping regions of the two helical structures by the inner fabric helix. The gap between the outer PVDF helix and the core formed by the inner fabric helix causes the outer PVDF helix to easily rotate along the pulling direction during the stretching motion, which results in a highly stretchable helical device.

In Figure 1b, when a force *F*_H_, which is a stretching force acting along the longitudinal direction to the core, is applied to the device, the tensile stress on the piezoelectric strap in the twined direction can be expressed as

(1) $$T_{1\;}=\frac{\left(F_{H}-k_{c}\Delta l_{H}\right)\sin\theta \;}{wh}\;$$

where *k*_c_ is the effective spring constant of the structure formed by the elastic core and the two helical straps around it, *Δl*_H_ is the change in length equal to the difference in length relative to its original length, θ is the winding angle of the PVDF strap, and *w* and *h* are width and thickness of the PVDF strap, respectively. The polarization along the thickness direction of the PVDF strap is given by *D*_3_ = *T*_1_*d*_31_, where *d*_31_ is the piezoelectric coefficient; therefore, the generated charge can be calculated by surface integration of the electric flux density as follows:

(2) $$q\left(t\right)=\int_{0}^{w}\int_{0}^{a}D_{3}dl=\int_{0}^{w}\int_{0}^{a}T_{1}d_{31}dl\;$$

where *a* is the arc length of the PVDF strap. For the helical structure, the arc length of the PVDF is given by

(3) $$a=\sqrt{{\left(2\pi rn\right)}^{2}+{\left(l_{0}+\Delta l_{H}\right)}^{2}}=\frac{l_{0}+\Delta l_{H}}{\sin\theta }$$

where *n* is the number of turns, and *r* and *l*_0_ are the radius and initial length of the helical spring, respectively. As the winding angle increases, the number of turns and total arc length of the PVDF strap decreases for a fixed initial length *l*_0_ and initial radius *r*_0_.

**Figure 2:**
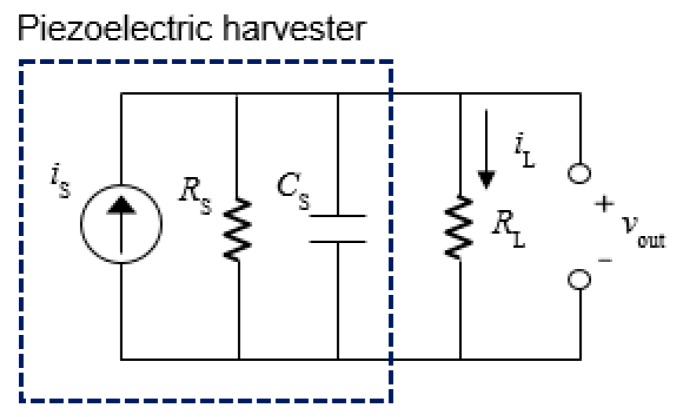
Equivalent model of the piezoelectric energy harvester with a resistive load.

By substituting *T*_1_ in Equation (1) and *a* in Equation (3) into Equation (2) and with the assumption of uniform stress throughout the PVDF film along the width and length, the charge generated by the displacement can be expressed as

(4) $$q\left(t\right)=\frac{\left(F_{H}-k_{c}\Delta l_{H}\right)\sin\theta \cdot a}{h}\;d_{31}=\frac{F_{l}\cdot \sin\theta \cdot a}{h}\;d_{31}\;=\frac{F_{l}\cdot a\;}{h}\;d_{31}\sqrt{\frac{{\left(l_{0}+\Delta l_{H}\right)}^{2}}{{\left(2\pi rn\right)}^{2}+{\left(l_{0}+\Delta l_{H}\right)}^{2}}}$$

where *F*_l_ is the longitudinal force applied on the PVDF strap. A piezoelectric element can be represented as a source current *i*_s_ in parallel with its internal capacitance, as shown in Figure 2 [29]. When a time-varying strain is applied on the piezoelectric material, the source current can be obtained by taking a time differentiation of the charge in Equation (4), which can be expressed as

(5) $$i_{s}=\frac{dq\left(t\right)}{dt}=\frac{d}{dt}\left(\frac{F_{l}\cdot a\;}{h}\;d_{31}\sqrt{\frac{{\left(l_{0}+\Delta l_{H}\right)}^{2}}{{\left(2\pi rn\right)}^{2}+{\left(l_{0}+\Delta l_{H}\right)}^{2}}}\right)$$

where both the radius *r* and change in length *Δl*_H_ are time-dependent variables during the actuation. When core diameter reduces linearly with the longitudinal force on PVDF strap, the source current can be further expressed as

(6) $$i_{s}=\frac{d}{dt}\left(\frac{k_{c}\Delta l{\left(l_{0}+\Delta l_{H}\right)}^{2}\;a}{2\pi nh\sqrt{a^{2}-{\left(l_{0}+\Delta l_{H}\right)}^{2}}}\;d_{31}\frac{1}{\sqrt{{\left(2\pi rn\right)}^{2}+{\left(l_{0}+\Delta l_{H}\right)}^{2}}}\right)$$

where *k*_c_ is the proportional constant representing the elasticity of the core in the transverse direction.

**Figure 3:**
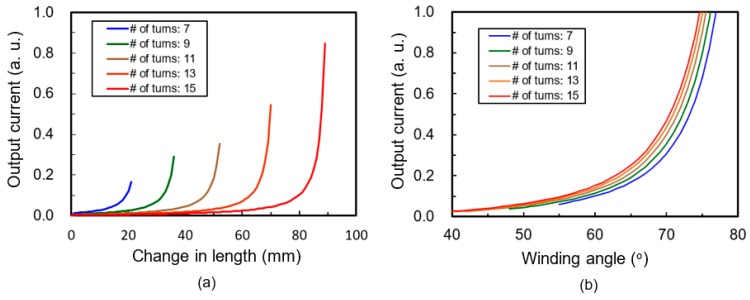
Theoretically calculated output current in arbitrary units (a.u.) for various (**a**) stretched changes in length and (**b**) winding angle variations.

When the device is stretched at constant velocity, the magnitude of the source current can be calculated from Equation (6) and plotted in Figure 3 in arbitrary units (a.u.) for various numbers of turns. In these graphs, the initial length and radius of helical structure are fixed at 110 mm and 1.8 mm, respectively. Figure 3a is a plot of the generation current for various changes in length when the device is stretched. As the stretched length increases, the tensile stress in the PVDF strap increases, and consequently, more charges are generated during the operation. For a given HPEH with fixed arc length and number of turns of helix, the winding angle increases as the device is stretched. Therefore, the generation current can also be calculated using the winding angle as a variable, as shown in Figure 3b. The horizontal axis in this figure is the winding angle of the PVDF strap, which increases with the stretching length. This graph shows that the generation currents increase more sharply when the winding angles are more than around 70°, regardless of the initial winding angles or the number of turns. From the equivalent model of the HPEH with a resistive load in Figure 2, the output voltage (*V*_out_) and power (*P*_o_) transferred to the resistive load can be calculated as

(7) $$V_{\mathrm{out}}\left(t\right)=i_{L}\left(t\right)R_{L}=i_{s}\left(t\right)R_{L}\sqrt{\frac{R_{s}^{2}+X_{s}^{2}}{{\left(R_{s}+R_{L}\right)}^{2}+X_{s}^{2}}}$$

(8) $$P_{o}\left(t\right)=i_{L}^{2}\left(t\right)R_{L}=i_{s}^{2}\left(t\right)R_{L}\frac{R_{s}^{2}+X_{s}^{2}}{{\left(R_{s}+R_{L}\right)}^{2}+X_{s}^{2}}$$

where *i*_L_ is the output current on the load resistor, and *R*_s_ and *X*_s_ are the source resistance and reactance of the harvester device, respectively [29].

In addition, these numerical calculations tells that the generation current increases as the number of turns decreases, namely, as the initial winding angle increases under given stretched length. As the number of turns decreases and the winding angle increases, more tension is applied along the direction longitudinal to the PVDF strap. However, as the PVDF film itself is not an elastic or a stretchable material, the maximum stretchable length is restricted by the PVDF strap and not by the elastic core. For example, if the number of turns is seven in our design, the maximum change in length is just about 20 mm in theory, as shown in Figure 3. An energy harvester will not be appropriate for a garment application unless it provide a certain degree of elasticity. If the HPEH is less elastic than the fabric of the garment, the stretching force induced by the human body motion is not effectively transferred to the HPEH resulting in a low output power. Therefore, there has to be a trade-off between the harvested output power and maximum elasticity for determining the number of turns of the PVDF helical structure. In this work, the number of turns is fixed at 11 so that the elasticity of HPEH becomes similar to that of the garment fabric used herein. 

In addition to the number of turns, the core diameter and the width of the PVDF strap and the fabric band are also important parameters in the design and application of HPEHs. In this work, three different types of HPEH are designed as shown in Table 1. The core diameter is 5 mm for Type 1 and 3 mm for Type 2 and Type 3. The width of the fabric band is fixed at 5 mm, while the width of the PVDF strap is 5 mm for Type 1 and Type 2 and 3 mm for Type 3. The relatively large dimensions of the cores and PVDF straps were determined for easy but precise manual implementation of the preliminary design of our proposed harvester.

## 3. Fabrication

**Figure 4:**
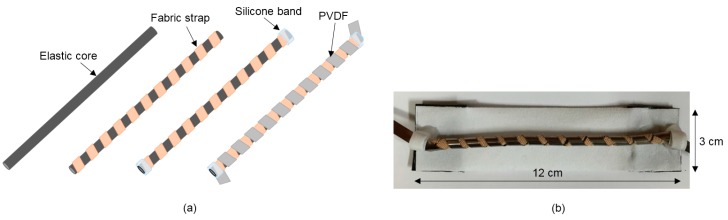
(**a**) Fabrication process of the helical piezoelectric energy harvester. (**b**) Photograph of a single helical piezoelectric energy harvester (HPEH) fixed on an elastic fabric.

Figure 4a displays the fabrication process of the HPEH. First, a fabric band (Benefact SX-4113, Nippon Sigmax Co., Tokyo, Japan) is twined on an elastic core to form a helical shape around the core. Next, a PVDF strap is twined in the opposite direction on the fabric band. A 110-μm-thick PVDF sheet (TE Connectivity Co., Berwyn, PA, USA), with a Ni/Cu alloy deposited on both sides as electrodes, is used as the piezoelectric helix. The piezoelectric coefficients *d*_33_ and *d*_31_ of the PVDF are 33 pC/N and 23 pC/N, respectively. No adhesives were used between the core and the fabric band or PVDF. Therefore, the HPEH is completed by fixing both ends of the helical PVDF and fabric straps to the core by using a silicone patch and cotton bandages. This device is tested to characterize the performance of a single HPEH. 

**Figure 5:**
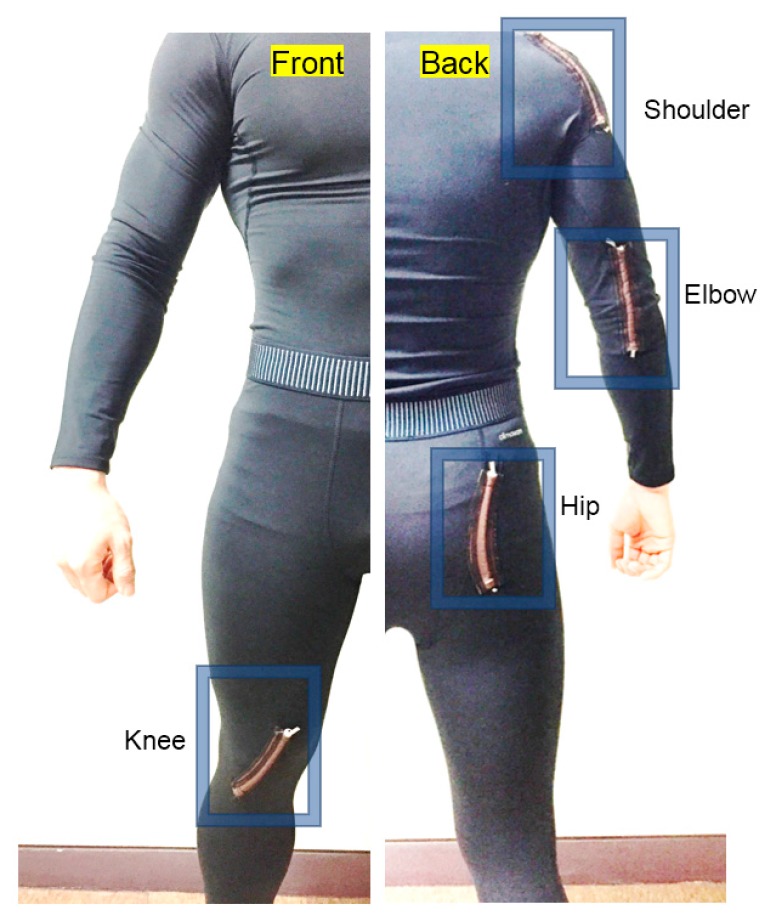
Photographs of the front (**left**) and back (**right**) views of the wearable energy harvesting garments.

In addition, the HPEH is embedded in a garment to investigate its operational performance and output properties with actual human motions. For this purpose, the HPEH is fixed on an elastic fabric as shown in Figure 4b. Here, it is desirable that the elasticity of the fabric is similar or higher than that of the HPEH and the garment. For human-wearable application, the HPEHs are embedded at four positions in the garments—shoulder, elbow, hip, and knee—by stitching them using elastic threads as illustrated in Figure 5. A tight-fitting but elastic shirt (Techfit S19455, Adidas, Seoul, Korea) and pants (Techfit D82122, Adidas) are used as test garments. The four positions where the HPEHs are embedded are the joint regions so that the energy is generated from general human body motions.

## 4. Results and Discussion

### 4.1. Single HPEH

**Figure 6:**
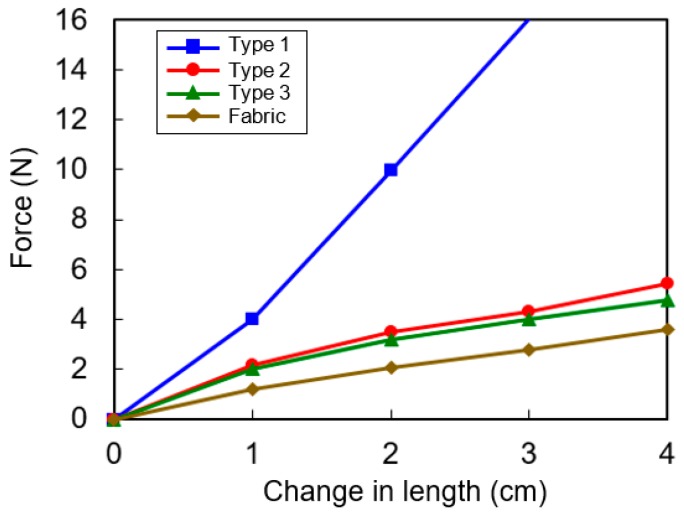
Pulling force versus change in length of the fabricated devices and garment fabric.

The elasticity of the garment fabric used in this work and the two types of HPEHs having different PVDF widths were experimentally investigated. The changes in length of the samples were measured while applying stretching forces. The stretched length linearly increases with the applied force as shown in Figure 6. The spring constant is measured as 550 N/m and 125 N/m for Types 1 and 2, respectively. The spring constant of the garment fabric is about 80 N/m, therefore, Type 2 is suitable for garment applications. If the spring constant of the HPEH is much larger than that of the garment fabric, the efficiency of power generation will be poor because most of the strain will act on the fabric and not on the HPEH during the body motions. In this experiment, the initial length of the fabricated device is 12 cm and the maximum change in length that is possible by stretching without damage is around 7 cm. As a result, the stretch ratio, i.e., the length after stretching divided by the initial length, is 158%.

**Figure 7:**
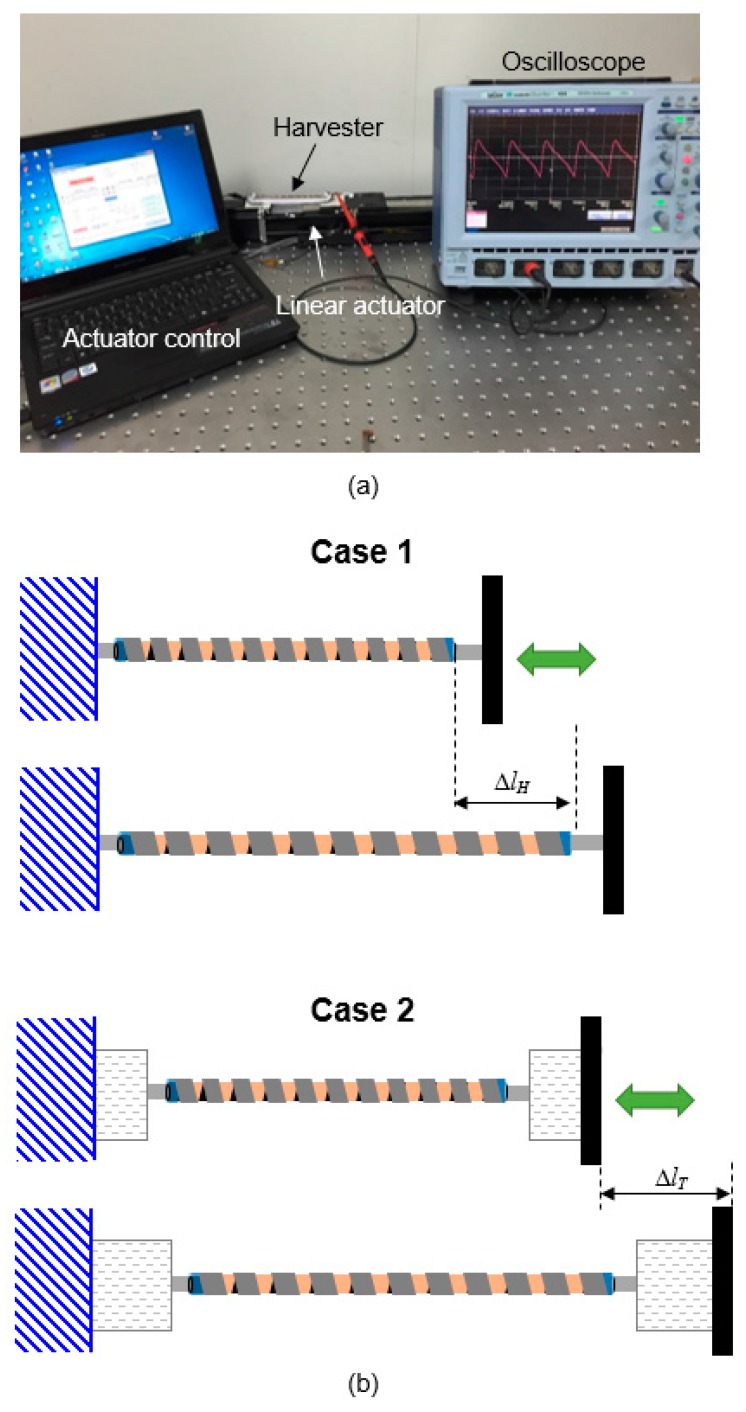
(**a**) Measurement setup. (**b**) Two configurations of the experiment. In Case 1, a helical piezoelectric energy harvester is directly connected to the actuator, while in Case 2, the device is connected to the actuator through fabric pieces.

Figure 7a displays the measurement setup of a single HPEH device. The output is measured when a force is applied to the fabricated device using a PC-controlled linear actuator (SOL-045BLM, Soltech Co., Ansan-si, Gyeong gi-do, Korea). The operation frequency is fixed at a low value of 1 Hz considering the slow human body motion. The output voltage is measured with an oscilloscope (Wavesurfer 454, Lecroy, Chestnut Ridge, NY, USA). 

The output was measured for two cases. In Case 1, the fabricated HPEH is directly connected to the linear actuator to apply the tensile force as shown in Figure 7b. In this case, the displacement of the actuator is equal to the change in length of the HPEH. In Case 2, fabric pieces of the garment are connected to both ends of the HPEH. Then both the fabrics and the HPEH are stretched when the actuator pulls the end of the fabric as depicted in the figure. Consequently, in this case, the change in length of the HPEH is smaller than the displacement of the actuator. Case 2 can be modeled as a series connection of two elastic springs with *k*_H_ and *k*_f_, which are the effective spring constants of the HPEH and fabric pieces, respectively. When the HPEH is stretched through the fabric pieces in Case 2 with a constant force *F*_T_ (which is a constant force mode), the force applied on the HPEH is equal to *F*_T_. On the other hand, in the constant change in length mode, the total change in length Δ*l*_T_ is driven by the actuator. Then, the force applied on the HPEH is expressed as

(9) $$F_{H}={\left(\frac{1}{k_{H}}+\frac{1}{k_{f}}\right)}^{-1}\Delta l_{T}$$

Therefore, the force on the HPEH increases with the spring constant of the HPEH *k*_H_, regardless of the operation mode. As *k*_H_ increases relative to *k*_f_, the force on the HPEH rapidly increases until *k*_H_ becomes equal to *k*_f_ in the constant change in length mode. When *k*_H_ is larger than *k*_f_, *F*_H_ increases relatively slowly with *k*_H_. As shown in Equations (1) and (5), the generation charge and resultant output power is proportional to the force on the PVDF strap *F*_l_. Therefore, *k*_H_ should be high while *k*_c_ should be small to increase the output power. *k*_c_ can be made small by using an elastic or thinner core material. However, because *k*_H_ and *k*_c_ are not independent variables, but closely related and influenced by various design parameters such as winding angle, core diameter, PVDF width, and length of the HPEH, the optimal design parameters should be determined via multi-physics modeling with finite element methods in future works. In addition, *k*_H_ cannot be increased too high because the garment will provide an uncomfortable fit to the human body. Therefore, a possible design guideline for the HPEH for a garment application is to make *k*_c_ as small as possible while maintaining *k*_H_ to as close as possible to *k*_f_.

**Figure 8:**
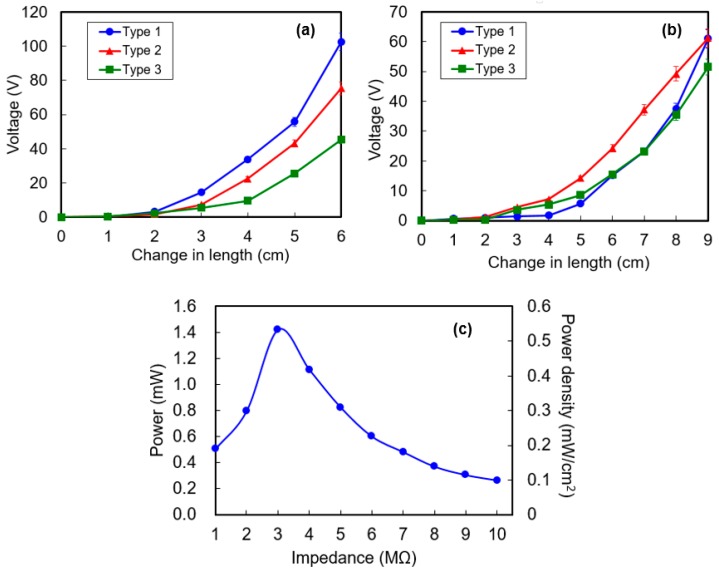
Open-circuit peak-to-peak output voltage while applying stretching force to obtain various changes in length for (**a**) Case 1 and (**b**) Case 2 experiments. (**c**) Peak output power across various load resistances for the Type 2 helical piezoelectric energy harvester measured under the condition of Case 1 experiment.

Figure 8a,b present the open-circuit peak-to-peak output voltage obtained from Cases 1 and 2 experiments, respectively while applying the stretching force to obtain various changes in length. The output voltage increases with the change in length because the stronger the tensile stress is applied on PVDF strap as the stretched length increase. In Case 1, the maximum output voltage for a change in length Δ*l*_H_ of 6 cm is 102 V, 75 V, and 45 V for Types 1, 2, and 3, respectively. Type 1 HPEH showed the highest output voltage compared to Type 2 and 3 because the total surface area of the PVDF helix is largest for Type 1 with 5 mm core diameter and 5 mm width. However, in Case 2, the output voltage from Type 2 is highest at a given change in length Δ*l*_T_ as shown in Figure 8b. In this case, the actual change in length Δ*l*_H_ of the HPEH is longer for Type 2 because it has a lower spring constant than Type 1. Therefore, because the output voltage depends only on the change in length of the HPEH and not on the entire change in length including fabric pieces, the output voltage from Type 2 was larger than that from Type 1. When the HPEH is embedded in a garment, both the HPEH and the fabric around it are under a tensile stress and are stretched. Therefore, in our work, the performance was measured for the garment with Type 2 HPEHs only even though changes in length during human body motions were observed for garments having both Types 1 and 2 HPEHs, as will be described in the following section. 

We measured the output power at various load resistances to investigate the optimum load impedance value and maximum output power. Figure 8c shows the peak output power across various load resistances for the Type 2 HPEH measured during the condition of Case 1 experiment. In this experiment, the stretched change in length was fixed at 6 cm and the operation frequency was 1 Hz. The maximum output power was 1.42 mW at a 3 MΩ load resistance, which corresponds to a power density of 0.42 mW/cm^3^ considering the volume of a single HPEH. The theoretical optimal load resistance of the Type 2 HPEH with estimated source capacitance *C*_S_ = 1 nF is about 160 MΩ at 1 Hz operation frequency, which is much larger than the experimental result. Since the resistance of the oscilloscope probe is 10 MΩ, the experimental load resistance value for maximum power transfer would be monitored at below 10 MΩ, when the operation frequency is very low as in the case of our experiments. In addition, any possible degradation of source resistance and other parasitic impedance might cause a difference between the theoretical and measured values; however, further examination is required to determine the exact causes including non-sinusoidal reciprocal stretching motion and the effect of extremely low frequency movement.

### 4.2. Energy Harvesting Garments

**Figure 9:**
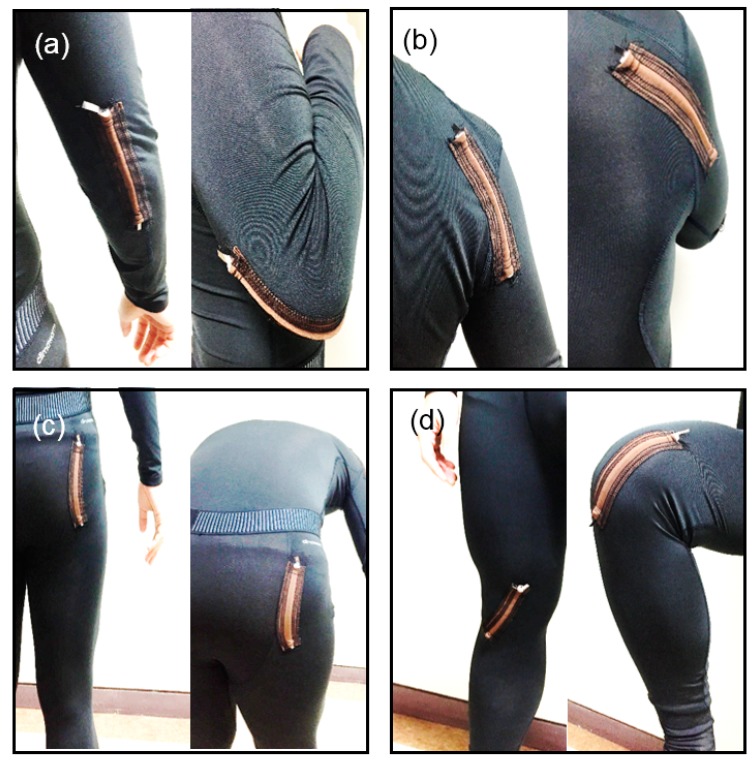
Flexion and extension motions at the four positions of (**a**) elbow; (**b**) shoulder; (**c**) hip, and (**d**) knee on application of the maximum bending condition.

**Figure 10:**
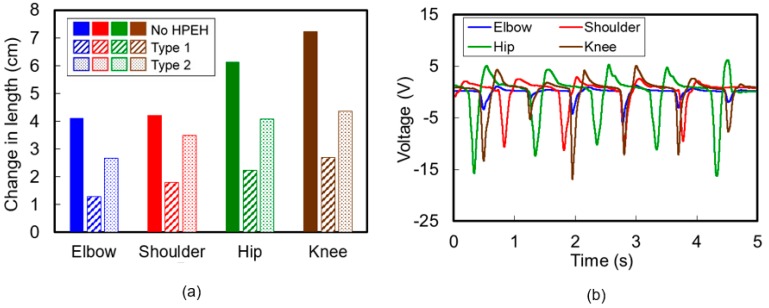
(**a**) Maximum change in length of the helical piezoelectric energy harvesters embedded in the garment. (**b**) Output voltage waveforms generated from the helical piezoelectric energy harvesters located at four positions.

In accordance with the description in the previous section, 12 cm-long HPEHs were fixed at four positions on the garments—shoulder, elbow, hip, and knee—to characterize the performance or the output properties during human body motions. After wearing the prepared garments, we measured the stretched changes in length and consequent output voltages when applying the maximum bending condition to the HPEHs at the four positions. Figure 9 shows the flexion and extension motions at the four positions of (a) elbow, (b) shoulder, (c) hip, and (d) knee. During these motions, the maximum changes in length of the HPEHs embedded in the garment were measured by using a flexible ruler and are depicted in Figure 10a. It can be seen from the figure that larger strains are induced in Type 2 HPEHs than in Type 1 at all four positions. Maximum change in length occurs at the knee, where the stretch ratio of HPEH is 141%.

To measure the output voltage during maximum stretching motion, the subject wearing the garments was asked to repeat each flexion and extension motion shown in Figure 9 while maintaining the range of motion and motion frequency as constant as possible. No special safety equipment is necessary for the participant because the total amount of charge and output voltage is not so high and the device is fully isolated from the body skin owing to the use of a thick fabric of cloth. Figure 10b displays the voltage waveforms generated from the Type 2 HPEHs located at four positions. These experiments show that the magnitude of the output voltage from the HPEH in a garment increases as the stretching length increases. The maximum peak to peak voltage was larger than 20 V at the knee. 

**Figure 11:**
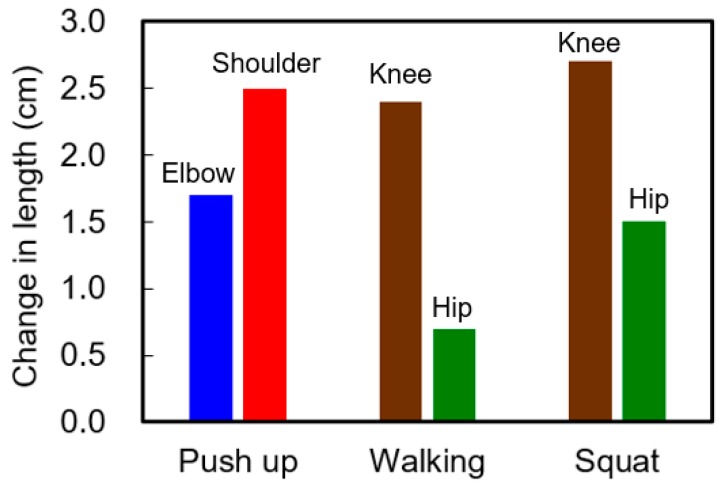
Change in length of each helical piezoelectric energy harvester in the garment during push up, walking, and squat.

In addition to the characterization of the energy harvesting garments during the intended maximized body motions, in this work, we measured the output during three daily activities of human body such as walking, push-ups, and squats. Figure 11 shows the stretched change in length of each HPEH in the garment during each of these motions. The test is performed by using the garment with Type 2 HPEHs. Compared to the changes in length of HPEHs during maximized motions in Figure 10a, it can be seen that the change in length on each HPEH during these three typical motions is relatively small. During the push-up motion, the change in length of the HPEHs is 1.7 cm and 2.5 cm at the elbow and shoulder, respectively. The HPEHs on the knee and hip are not stretched during the push-up motion, but are stretched during walking and squatting motions. The change in length at the knee is maximal as 2.4 cm and 2.7 cm for the walking and squat motions, respectively. The stretching on the hip is relatively small compared to the other joints. Maximum change in length on the hip is obtained during squatting motion, which is 1.5 cm as shown in the graph. 

**Figure 12:**
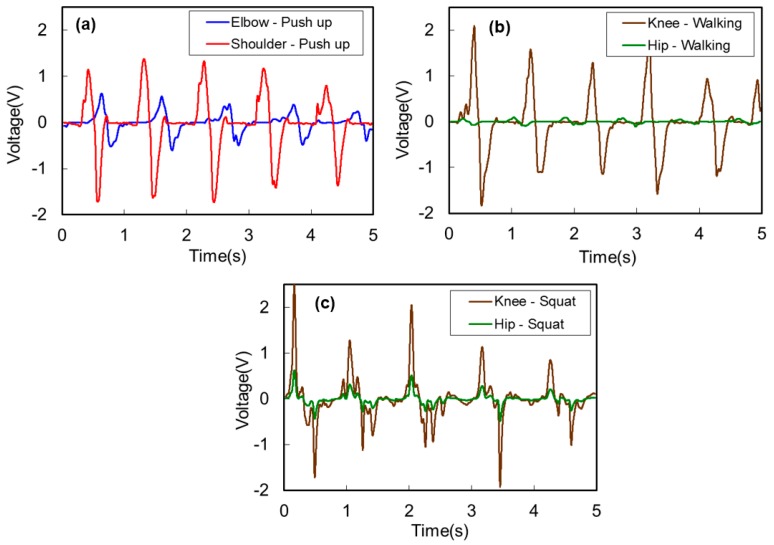
Output voltage waveforms from the helical piezoelectric energy harvesters during the three body motions of (**a**) push-up, (**b**) walking, and (**c**) squat.

Figure 12 shows the output voltage waveforms from the HPEHs during the three body motions of (a) push-up, (b) walking, and (c) squat. Similar to the previous experiments with intended maximum stretching motions, magnitude of the output voltage depends on the stretched length in these experiments also. The maximum voltages that can be obtained from the energy harvesting garments are about 3.9 V at the elbow, 3.1 V at the knee, and 4.4 V at the knee during push-up, walking, and squat motions, respectively.

**Figure 13:**
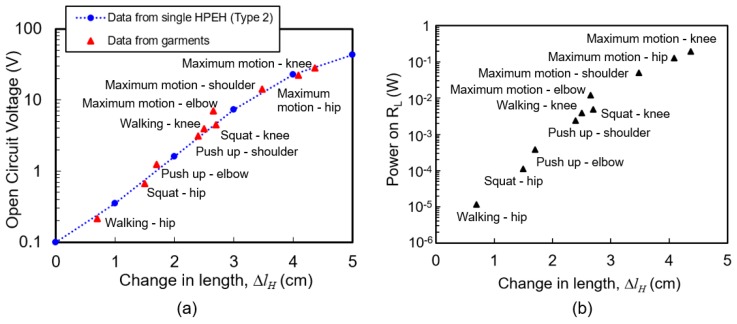
(**a**) Relation between stretched change in length and output voltage. Blue dashed line: Voltage versus change in length from Case 2 experiment with Type 2 single helical piezoelectric energy harvester (HPEH) device obtained from Figure 5 and Figure 6. Red triangular dots: Maximum open circuit voltage versus change in length of HPEH on garments extracted from Figure 10, Figure 11 and Figure 12. (**b**) Power at 3 MΩ load resistance calculated from the open circuit voltage values.

The relation between the stretched change in length and output voltage of the proposed energy harvesting garments is compared with the response curve of the single HPEH in Figure 7 and Figure 8. In Figure 13a, the blue dashed line shows the voltage versus change in length in Case 2 experiment with Type 2 single HPEH device. The maximum voltage and change in length data from the garments is extracted from the experiments in Figure 10, Figure 11 and Figure 12, which is depicted as red triangular dots. It can be seen that the output voltage does not vary linearly with the stretched length, though overall it increases as the stretched length increases. When the change in length is small (under 20% of its initial length), the output voltage is very small because most of the pulling force is applied to the elastic core of the HPEH and the tensile stress on the PVDF film is very small. However, when the change in length is over 30% of its initial length, the output is quite large and increases linearly with the stretched length. In this range, majority of the pulling force is applied to the helical PVDF because of the increased winding angle. In addition, it is clear that the data from the garment experiment, depicted as red dots, corresponds well with the unit HPEH experiments presented as the blue dashed line in the figure. The power at 3 MΩ load resistance in Figure 13b was calculated from the open circuit voltage values. It can be shown that a maximum output power of 0.2 mW is possible from the energy harvesting garment during knee motion.

## 5. Conclusions

In this study, we designed energy harvesting garments by applying helical piezoelectric energy harvesters to stretchable tight-fitting sportswear. After theoretically analyzing the design parameters that affect the output performance, an experimental demonstration was performed using unit helical harvesters with three different structural dimensions. The fabricated harvester was highly elastic and could be stretched up to 158% of its initial length. The maximum output power was obtained from the Type 2 device, which was as high as 1.42 mW or 296 μW/cm^2^ in power density at an operating frequency of 1 Hz and a load resistance of 3 MΩ. This value is comparable to the output power density of 96 μW/cm^2^, 125 μW/cm^2^, 5.10 μW/cm^2^, and 170 μW/cm^3^ reported in previous literature using piezoelectric polymer materials [24,29,31,33]. From an energy harvesting garment with four helical harvesters, maximum output power was obtained from the knee. The peak-to-peak output voltage from the harvester on the knee was larger than 20 V during maximum bending motion and 4 V during squat motion. The output voltage was considered to be inadequate for practical applications when the stretching ratio was below 20%. However, our research results demonstrated that reasonable output voltages over 3–5 V can be obtained from the proposed energy harvesting garment during daily human motions such as walking, push-ups, and squatting.

## Figures and Tables

**Table 1:** Parameters of the three types of harvesters.

Parameters	Type 1	Type 2	Type 3
Elastic Core Diameter	5 mm	3 mm	3 mm
PVDF Width, *w*	5 mm	5 mm	3 mm
Number of Turns, *n*	11	11	11
Initial Winding Angle, θ_0_	~ 30°	~ 40°	~ 30°
